# Machine Learning in Microwave Medical Imaging and Lesion Detection

**DOI:** 10.3390/diagnostics15080986

**Published:** 2025-04-12

**Authors:** Wenyi Shao

**Affiliations:** EMAI LLC, Laurel, MD 20723, USA; wshao@ieee.org

**Keywords:** deep learning, lesion detection, machine learning, microwave medical imaging

## Abstract

Machine learning (ML) techniques have attracted many microwave researchers and engineers for their potential to improve performance in microwave- and millimeter-wave-based medical applications. This paper reviews ML algorithms, data acquisition, training techniques, and applications that have emerged in recent years. It also reviews state-of-the-art ML techniques applied for the detection of various organ diseases with microwave signals, achieving more successful results than using traditional methods alone, such as a higher diagnosis accuracy or spatial resolution and significantly improved efficiency. Challenges and the outlook of using ML in future microwave medical applications are also discussed.

## 1. Introduction

In recent years, advances in medical technology have highlighted the potential of microwave medical imaging (MMI) for non-invasive disease detection [1,2,3,4,5,6,7,8], including COVID-19 [9]. MMI leverages the differences in electromagnetic wave propagation characteristics across various tissues to generate internal images of the human body. This technology has shown promise, particularly in the early detection of conditions such as breast cancer and stroke. Compared to traditional imaging methods like X-ray CT [10], ultrasound [11], and MRI [12], microwave imaging offers advantages including lower costs, higher safety (as it avoids ionizing radiation), and enhanced soft tissue differentiation. Consequently, there is growing interest in harnessing microwave imaging for accurate and non-invasive medical diagnoses.

However, MMI faces significant challenges in signal processing and image reconstruction due to the inherent complexity of microwave signals in biological tissues. As microwaves propagate through tissues, they encounter effects such as multipath propagation, scattering, and attenuation [13,14,15], complicating the reconstruction of clear, accurate medical images from the received signals. In recent years, machine learning (ML) techniques were introduced to enhance the performance and accuracy of medical imaging systems [16,17,18,19], including microwave medical imaging. Compared to conventional approaches [20,21,22], ML techniques are able to generate higher-accuracy images and results, leading, for instance, to higher diagnostic accuracy or spatial resolution, as well as significantly improved efficiency. Deep learning (DL) methods, in particular, have garnered attention for their remarkable performance in image recognition and signal processing, making them suitable for addressing the complex problems posed by microwave imaging.

The application of ML in MMI not only facilitates the handling of large datasets but also enhances the system’s capability to identify and detect diseases. For instance, deep neural networks can automatically detect and classify areas of concern, significantly reducing the likelihood of human error. Moreover, ML methods play a key role in optimizing image reconstruction algorithms, mitigating noise, and improving image resolution. Overall, ML provides novel solutions to challenges in MMI, accelerating its advancement and applications in medical diagnostics.

ML is extensively used for classification tasks, where algorithms are trained to categorize data points based on underlying patterns. In the medical field, ML significantly enhances diagnosis, prognosis, and treatment planning. Techniques such as support vector machines (SVMs), decision trees (DTs), random forests (RFs), and logistic regression (LR) are commonly employed to classify diseases, predict patient outcomes, and identify risk factors. In microwave medical imaging, ML models have been applied to classify microwave images or signals to detect conditions like cancer or fractures, analyze patient data to predict readmission likelihood, and identify abnormal patterns in vital signs. By learning from large datasets, these models improve accuracy and efficiency, enabling healthcare professionals to make more informed decisions and facilitating earlier interventions for better patient care.

Compared to other reviews on artificial intelligence adopted in microwave imaging and microwave technologies, this article contributes a narrow review solely focused on biomedical applications. We systematically summarize the most recent advancements over the past few years in applying machine learning (ML) to microwave medical imaging and disease detection. We structure the paper based on the role of ML algorithms. This includes traditional classification and regression models, disease monitoring, and their specific contributions to microwave signal processing and image analysis. Under each specific role, discussions are expanded by various organ-related diseases such as the brain, the breast, the knee, the torso, etc. Finally, we explore future trends, highlighting the challenges and opportunities for ML in advancing microwave medical imaging.

## 2. Lesion Classification

### 2.1. Brain

Hossain et al. developed a lightweight neural network (NN) to classify reconstructed brain images into six categories: no tumor, single benign tumor, single malignant tumor, double benign tumors, double malignant tumors, and a combination of a single benign and a single malignant tumor [23]. The training dataset containing 920 samples was generated using a nine-sensor microwave brain imaging system with a fabricated tissue mimicking brain phantom. Malignant tumors, with irregular elliptical or triangular shapes, and benign tumors, with a roughly round form, were positioned in various locations within the phantom. The developed NN achieved a classification accuracy of 96.97%, outperforming nine other NN models. Around the same time, the team developed another NN model for tumor segmentation using the same imaging system and dataset [24]. More recently, they applied transfer learning to the InceptionV3 model—a pretrained, state-of-the-art NN—further improving classification accuracy compared to their previous model [25].

Beyond brain tumor recognition, ML has also been applied to stroke diagnosis. Gong et al. developed a stroke classification algorithm using a decision tree (DT) method [26]. Their training dataset was collected using a wearable device equipped with eight antennas for 200 tests on a brain phantom which was divided into five subregions: front left, front right, back left, back right, and middle. The model aimed to determine the stroke’s location and classify it as either an intracranial hemorrhagic stroke or an ischemic stroke. In India, researchers explored various ML methods to specifically identify brain hemorrhages [27] in three potential locations: left subdural, right subdural, and intracerebral. Using a microwave dataset of 80 examples collected from a two-antenna system, their study found that DT, k-nearest neighbor (KNN), and random tree (RT) classifiers outperformed support vector machine (SVM) and multilayer perceptron (MLP) NNs in classification accuracy. Notably, both research teams analyzed and classified S-parameter data, in contrast to Hossain et al., who processed reconstructed images.

Other than tumor and stoke diagnosis, Ullah et al. developed a DL approach to classify the stages of Alzheimer’s disease (AD) as healthy, mild, or severe using reflected microwave signals [28]. Specifically, the study analyzed S_11_ parameters captured by antennas. A total of 200 frequency points, ranging from 20 MHz to 3 GHz, for each of the nine brain phantoms were recorded in complex form but converted to absolute values before being fed into the neural network. To account for variations in head sizes, simulations were conducted using multiple phantoms with different mesh-cell compositions. The proposed algorithm integrated a convolutional neural network (CNN) with a gated recurrent unit (GRU), achieving a classification accuracy of 87%. This DL-based method, which directly processed raw microwave data, demonstrated significant potential for early-stage AD monitoring.

### 2.2. Breast

**Figure 1:**
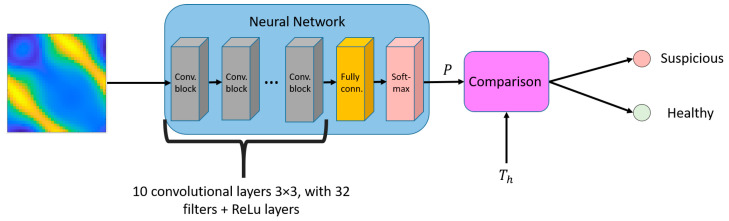
Scattering signal input into an NN to identify whether a patient is healthy or cancer suspicious [31].

Microwave breast imaging is another area of growing research interest worldwide. The application of machine learning (ML) for breast lesion analysis dates back to 2007 when El-Shenawee proposed a fully connected NN to predict the presence or absence of a tumor [29]. The training dataset consisted of 7000 with/without-tumor cases in a simplified breast phantom with varying tumor positions. Building on this work, Franceschini et al. employed a convolutional NN (CNN) to improve the classification of healthy and suspicious cases using multiple digital breast phantoms [30]. Similarly, researchers from the University of Manitoba applied CNNs to experimental data to predict tumor presence (Figure 1) [31], demonstrating the potential of ML in enhancing microwave-based breast cancer detection. The experimental dataset contained 1257 S-parameter scans of MRI-derived phantoms, including 249 monostatic scans of 13 phantoms and 1008 bistatic scans of 66 phantoms over 1–8 GHz, and is open to the public for researchers who have the same interest [32].

In 2018, Oliveira et al. introduced a microwave breast diagnosis system that utilized radio frequency signals to differentiate between benign and malignant tumors [33]. The training dataset was generated using finite-difference time-domain (FDTD) simulations, modeling scans of 72 tumor models in five different positions within three numerical breast phantoms [34], using a multistatic scanning system consisting of 12 antennas. Tumors were modeled in varying sizes, ranging from 6 mm to 20 mm in diameter, and categorized into two distinct shape groups: smooth-bordered tumors representing benign cases and spiculated-bordered tumors representing malignant cases. These tumors were placed in five different positions within the breast. The time-domain signals were then grouped based on antenna locations and analyzed by the classification algorithm. The study found that signals from the three antennas closest to the tumor provided the most accurate diagnostic results.

Differently, Mojabi et al. developed a CNN to classify breast tissues using reconstructed-image inputs in 2020 [35]. The input data consisted of complex-valued permittivity images from microwave imaging and compressibility and attenuation images from ultrasound, both obtained using the Gauss–Newton inversion algorithm. A three-stage U-Net was trained to classify each pixel into one of five categories—background, fat, skin, fibroglandular tissue, or tumor—effectively generating a segmentation map of the input image. A total of 400 breast images selected from eight 3D phantoms were split to two groups, with 350 for training and the remaining for testing. Additionally, the CNN produced an uncertainty map, highlighting the confidence level of its classification for each pixel.

Wang proposed a modified AlexNet for breast tissue classification with transfer learning [36] using 966 training images. In this approach, the main architecture of AlexNet was retained, while the final classification layer was customized and trained on a tailored dataset. The training dataset consisted of dielectric breast phantoms, representing the real and imaginary parts of complex-valued permittivity, derived from MRI images. All breast images were resized to 227 × 227 pixels, the standard input size for AlexNet. The model aimed to classify each breast image into one of five categories: fatty, dense, heterogeneously dense, very dense, and breasts containing tumors. The last category included two subgroups—fatty and very dense breasts with tumors. While the model achieved excellent results, successfully identifying all tumor-containing breasts, its impact was limited by the tumor modeling approach. Specifically, tumors were simplified as spherical shapes with only two fixed sizes (radii of 5 mm and 10 mm), reducing the model’s applicability to more complex tumor geometries.

MammoWave is a microwave breast cancer detection system currently undergoing clinical validation in several hospitals across Europe [37,38,39,40]. The system features a pair of air-coupled antennas that rotate around the breast, capturing 2D microwave signals reflected from breast tissues at various azimuthal positions. Recently, the developers implemented an SVM algorithm to classify patients with breast tumors directly from microwave response data collected at one of the clinical validation centers [41]. A total of 61 breast phantoms were used for training, validation, and testing. However, the model did not distinguish between benign and malignant tumors. The proposed ML model achieved an accuracy of 91%, a sensitivity of 84.40%, and a specificity of 95.50%.

In addition, ML has also been applied to breast microwave radiometry (MWR), a technique which detects breast cancer by analyzing the thermal properties of tissues. A neural network (NN) developed by a UK-led research team was trained to assess breast cancer risk levels based on MWR data [42]. The model was trained by 4912 cases with 4377 defined as low-risk and 535 as high-risk. The authors reported that an evolutionary algorithm-based random search strategy outperformed the gradient descent method in optimizing the NN weights.

Additionally, Turkish researchers developed an SVM-based model to classify rat breast tissues into three categories: healthy, benign, or malignant [43]. Their model analyzed the dielectric parameters measured for 635 normal breast tissue samples, 195 adenosis, and 350 malignant samples between 0.5 and 6 GHz, using an open-ended coaxial probe technique, and finally achieved a median classification accuracy of 94.4%.

### 2.3. Others

ML has been applied to various other microwave medical applications. A research group from China developed two ML models trained on a dataset from 15 subjects to assess acute tonic cold pain (CP) using microwave transcranial transmission (MTT) signals [44]. The objective was to leverage microwave scattering signals from the brain to more accurately evaluate neural activity associated with pain perception. Their findings indicated that the RF method was more reliable and accurate than the SVM in distinguishing between CP and no pain (NP) conditions.

In Italy, a DL approach was used for real-time temperature monitoring during hyperthermia treatment (HT) for neck tumors [45]. HT is an adjunctive cancer therapy that heats malignant tissues to destroy cancerous cells. Maintaining the correct therapeutic temperature is crucial—ensuring that the targeted tissue reaches the desired temperature while preventing overheating of surrounding healthy tissue. To facilitate this, real-time microwave imaging was used as a non-invasive temperature monitoring tool. The reconstructed microwave images were fed into a CNN, which classified the heating status into three categories—unheated, therapeutic, or hot—each corresponding to a specific temperature range. In total, 5000 sample images were simulated to provide the training data.

Another application of ML in microwave medical imaging is skin cancer diagnostics. A CNN-based system was developed to enhance microwave reflectometry for skin cancer assessment [46]. The system measured the dielectric properties of skin lesions (by S_11_ parameter data from 22 volunteers) at different frequencies and integrated dermatoscope images—optical images obtained using a handheld dermatological device. The CNN then classified the subject as either healthy or cancerous, improving the accuracy and efficiency of skin cancer diagnosis. Examples discussed in this section have been compared in Table 1.

## 3. Estimation and Monitoring

ML algorithms have been developed to estimate medical parameters for patient monitoring. These algorithms use regression models to generate numerical outputs, which can be visualized as probability maps or presented as standard medical parameters understandable to both doctors and patients.

**Figure 2:**
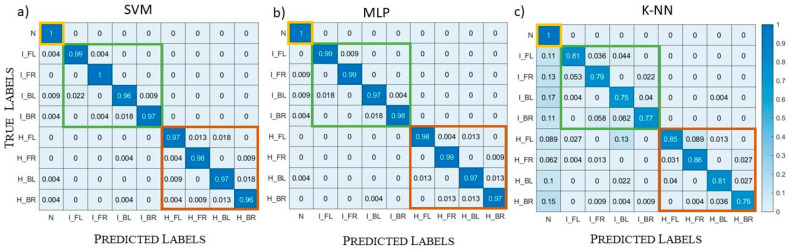
K-NN underperforms in brain stroke classification when compared to SVM and MLP [48].

One example is by Lai et al., who developed a DL model to estimate stroke location based on microwave signals [47]. Instead of performing full brain image reconstruction, as performed in inverse imaging, their approach identified specific grids with a high likelihood of stroke occurrence within a simplified oval representation of the brain. This oval image was divided into 356 grids, each measuring 8×8 mm, corresponding to probable stroke locations. This method resembles radar-based algorithms that highlight target locations but differs in that it does not reconstruct the actual head profile. Instead, it uses a standardized oval template applicable to various patients. A similar study was conducted by Mariano et al. [48], who tested three different classifiers—SVM, NN, and KNN (shown in Figure 2)—finding that both SVM and NN performed well, whereas KNN failed to classify test sets accurately.

Another ML application involves monitoring hydration levels in muscle tissue. A NN was developed to assess hydration status in a muscle phantom modeled by varying bovine serum albumin concentrations [49]. S-parameters were measured and analyzed using an Elman NN, achieving an estimation error of less than 3.3%.

Another interesting example is to monitor glucose level in blood for diabetes prevention using a microwave signal. A research group from Tianjin University, China, developed several NN models to precisely estimate blood glucose concentration using ultra-wideband (UWB) microwave signals [50,51]. S-parameters in the 0.2–4 GHz range were measured, preprocessed, and analyzed by NNs, which were validated on volunteer patients with high accuracy. Additionally, researchers from Canada and Europe proposed a microwave glucose sensor integrated with ML capabilities for continuous glucose monitoring in diabetic patients [52]. They developed a long short-term memory (LSTM) network to track glucose levels and predict future fluctuations in real time, using S_11_ amplitude as input. An anomaly detection algorithm was incorporated to mitigate environmental factors, enhancing prediction accuracy.

## 4. Image Reconstruction

### 4.1. Brain Imaging

**Figure 3:**
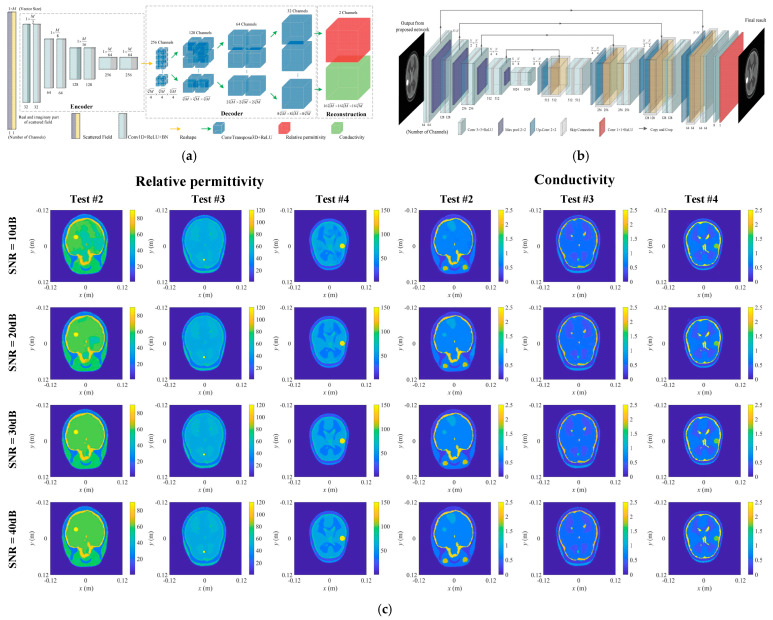
Reconstruction of an NN algorithm by Liu [55]: (**a**) image reconstruction CNN (FCERNN) structure; (**b**) the U-net for improvement; and (**c**) reconstructed images with anomalous scatters in presence.

Microwave brain imaging has primarily focused on brain tumor and stroke detection. In 2022, Liu’s team developed several ML algorithms for 3D super-resolution brain image reconstruction [53,54,55]. Each algorithm followed a two-step process: first, a NN—either a semi-fully connected NN or a CNN—performed a coarse reconstruction of both the relative permittivity and conductivity distribution simultaneously. This was followed by a U-Net to enhance image quality. The training dataset was generated from a single 3D dielectric brain model, which was rescaled to create variations in brain sizes, with abnormal scatterers randomly distributed within the model. Figure 3 illustrates the structure of the CNN used for reconstruction, the U-Net for image enhancement, and an example of a reconstructed image from one of their algorithms.

Abbosh’s team at the University of Queensland has developed multiple ML models for specific microwave brain imaging tasks, including image reconstruction [56], stroke classification [57], clutter removal [58], and lesion localization and assessment [59]. They have also introduced a graph attention network to integrate antenna array configurations directly into the NN structure [60]. Recently, the team proposed a U-Net-based model for reconstructing the dielectric properties of brain tissue using time-domain microwave signals. To address the challenge of limited clinical training data, they applied transfer learning: the U-Net was initially pretrained on a large dataset of geometric objects with random shapes to learn dielectric profile reconstruction and then fine-tuned using a limited labeled dataset of unhealthy brain scans. This approach aimed to mitigate data scarcity issues, as noted by the authors. Beyond brain imaging, Abbosh’s team has recently expanded their research to liver disease detection using ML algorithms applied to microwave signals [61].

### 4.2. MW Breast Imaging

Image reconstruction is a particularly challenging task for ML due to its highly nonlinear and often ill-posed nature. In 2020, Shao proposed a two-step training approach for learning-based reconstruction systems [62]. The first step involved training an autoencoder to compress complex object images into a compact representation. This simplified the second step, where a mapping was learned between microwave (MW) scattering signals and these compact representations. An end-to-end reconstruction system was then achieved by replacing the encoder with the trained learning system from step two and appending the decoder from step one. Building on Shao’s work, Kidera’s research team in Japan applied this approach to breast imaging [63]. They used 172 two-dimensional dielectric breast images from four patients as the training dataset, with data augmentation through rotations. While their system demonstrated successful validation on test data, the limited dataset impaired robustness, as autoencoders require a large population dataset to generalize well. In a subsequent study, they developed a fully connected NN trained in an end-to-end manner [64]. A key feature of this follow-up study was the preprocessing step: a fractional derivative algorithm [65] was applied to remove the skin response before feeding the scattering signal into the NN, improving the accuracy.

Baselice’s team also developed an end-to-end DL approach for microwave breast imaging [66]. They generated a virtual dataset of 2D breast phantoms through a procedural algorithm: first, an ellipse with axes ranging from 6.5 to 12 cm was generated in the center of the image. This ellipse was then randomly filled with fibroglandular, transitional, and adipose tissues [67], enclosed by a skin layer of 1.5–2.5 mm thickness. A total of 120,000 such profiles were generated, each discretized into 108 × 108 pixels. Thirty antennas were arranged in a circular formation (radius: 12 cm) around the breast, and scattering data were simulated using the method of moments [68]. The dataset—including both scattering data and breast profiles—was used to train a fully connected NN to reconstruct permittivity and conductivity maps of the breast. However, this study was limited by the simplification of breast profiles to ellipses and the absence of tumors in the training models. To address this, in a subsequent study, the team incorporated tumor models using the same data generation method and replaced the fully connected NN with a U-Net to improve image reconstruction [69].

**Figure 4:**
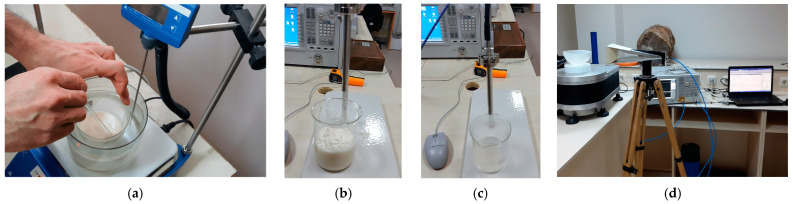
Phantom fabrication and measurement in [70]: (**a**) phantom fabrication; (**b**) healthy phantom; (**c**) tumorous phantom; and (**d**) measurement setup.

A similar approach was taken by Bicer [70], who developed both a fully connected NN and a deeper U-Net than Baselice’s for MW breast image reconstruction. The key distinction in Bicer’s work was the use of experimentally collected training data rather than purely synthetic data. The dataset was derived from measurements on 1000 fabricated breast phantoms, where mimic tissues were placed inside a custom beaker to create healthy and tumorous breast models (illustrated in Figure 4). While experimentally obtained data are often more clinically meaningful than simulated data, the fabricated phantoms in Bicer’s study were relatively simple and did not capture the full diversity of breast contours and sizes observed in real patients.

### 4.3. Neck Tumor Imaging

An NN-based microwave imaging approach for neck tumor detection was introduced by Dachena et al. [71]. This method aims to reconstruct the geometric and dielectric properties of the neck to identify potential tumors using scattered field data. A fully connected NN was trained on digital cross-sections of the neck, derived from a publicly available whole-body dielectric phantom. To simulate tumor cases, circular-shaped tumors were introduced into portions of the phantom. The dataset was further augmented by varying the dielectric and geometric properties of the original phantom to enhance generalization. The NN was optimized using a total of 30,000 generated phantoms, with 95% allocated for training and 5% reserved for testing. Examples discussed in this section have been listed in Table 2 for comparison.

### 4.4. Thermoacoustic Imaging

Thermoacoustic imaging (TAI) is a hybrid modality that utilizes microwave energy to induce ultrasound scattering from tissue, offering a promising diagnostic tool for various diseases. Applications of machine learning (ML) in TAI are conducted primarily by researchers from China.

In 2020, a researcher team from China explored the feasibility of using a deep CNN for detecting microwave-induced thermal lesions [72]. The study leveraged ultrasound backscattered signals to extract features associated with thermal lesions for detection and monitoring. A dataset comprising 1640 backscattered signal matrices from freshly excised porcine liver samples was used to train and test the CNN. The results demonstrated that the CNN framework effectively identified thermal lesions, showcasing its potential for improving the monitoring of cancer tissue ablation.

Additionally, Wang’s research team in China explored DL-enabled microwave-induced thermoacoustic imaging for applications in breast [73,74], brain [75], and blood vessel imaging [76]. In [76], they incorporated the realistic characteristics of ultrasound transducers into the NN design, which proved particularly effective for imaging elongated structures such as blood vessels. Unlike earlier studies that assumed omnidirectional transducer reception, this work accounted for the limited receiving angles of practical transducers, making the approach more applicable to real-world imaging scenarios.

## 5. Microwave Image Postprocessing

Using NNs, especially CNNs, to postprocess reconstructed medical images, such as segmentation and contrast improvement, has been widely explored in various imaging modalities. This section reviews key applications of CNNs for microwave image postprocessing.

LoVetri’s team developed a DL method to enhance thermoacoustic imaging for breast tumor detection [77]. When complex-permittivity images of the breast are reconstructed using the contrast source inversion (CSI) technique, clutter artifacts can obscure tumor detection. To address this issue, the team implemented a CNN with a U-net architecture to remove artifacts and improve tumor visibility. Expanding on this work, they later introduced a 3D U-net to process volumetric reconstructed images for the same purpose [78]. More recently, they developed an iterative segmentation approach that classifies breast tissue into fatty, transition, fibroglandular, and malignant regions [79]. This method enables a quantitative analysis of microwave images without requiring prior assumptions about dielectric property values, making it adaptable to various datasets and image qualities.

Additionally, Qin et al. proposed a multi-stream multi-task CNN (CNN_MM) for dual-modality imaging, integrating microwave and ultrasound data [80]. Unlike thermoacoustic imaging, where microwaves serve only as signal inducers, this dual-module system utilizes both microwave and ultrasound sensors to capture independent signals. The microwave and ultrasound contrast-source and field quantities, obtained via a backpropagation algorithm, were fed into a multi-input NN. By sharing the network, information from both modalities was synthesized to improve the imaging results. To handle multiple tasks, the network produced two outputs: one for the reconstructed physical parameter distributions (permittivity, ultrasound speed, and attenuation) and another for breast tissue segmentation.

In another example, Costanzo et al. developed an NN combining U-net and ResNet architectures for breast tumor segmentation [81]. The network was trained on three types of breast phantoms, categorized by density, with each dataset containing 500 samples. Unlike other studies reviewed in this article, which focus on segmenting all breast tissues, Costanzo’s approach specifically targeted tumor segmentation, refining the tumorous region and outputting it as the final network prediction.

## 6. Phantom Generation and Forward Computation

ML-based techniques require large training datasets, which, in medical imaging, means access to extensive patient data. However, obtaining such data is challenging due to privacy concerns and limited availability. Fabricating a large number of physical phantoms and collecting measurements in a laboratory setting is also impractical. Thus, leveraging generative models to create synthetic phantoms presents a promising solution for training ML algorithms.

**Figure 5:**
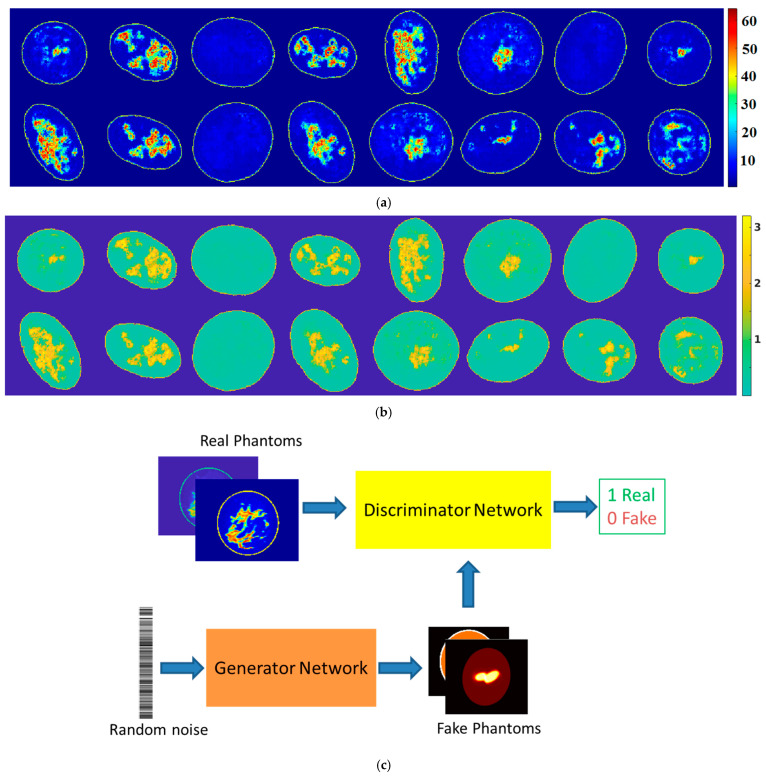
GAN for dielectric breast phantom generation [82]: (**a**) relative permittivity; (**b**) conductivity; and (**c**) training layout of GANs.

In 2022, a generative adversarial network (GAN) was developed to generate synthetic dielectric breast phantoms [82]. GANs consist of two competing networks—a generator and a discriminator—that are trained alternately. The generator produces synthetic data, while the discriminator learns to distinguish real data from generated samples. Through this adversarial process, the generator progressively improves until it can create realistic synthetic phantoms that resemble real patient data. The proposed GAN model was designed to generate both permittivity and conductivity phantoms of the same breast at a specific frequency. The training data derived from MRI-converted images from 34 patients, resulting in 1914 permittivity–conductivity image pairs. Figure 5 illustrates examples of GAN-generated permittivity and conductivity breast images at a single frequency. The paper also explored extending GAN models to produce broadband breast phantoms for multi-frequency or ultra-wideband (UWB) applications.

**Figure 6:**
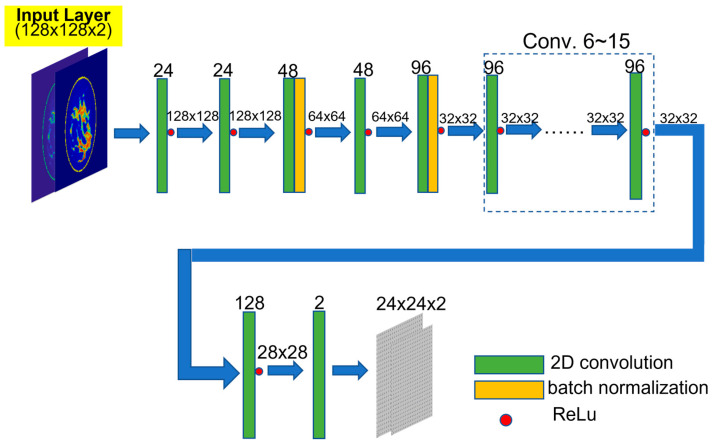
Estimating the scattering field of given breast phantoms at specified antenna positions (24 by 24) using CNN [83].

Following this work, the team developed a CNN, as shown in Figure 6, for microwave breast imaging simulations, specifically for forward computations [83]. Traditional electromagnetic near-field simulations are computationally intensive, often requiring hours or even days to complete. While parallel computing techniques using GPUs can accelerate these simulations [8], the cost and power requirements of high-performance GPUs remain a concern. The proposed CNN, trained by 20,000 synthetic breast phantoms generated by the GAN model, was able to predict scattered-field data at specific antenna positions, assuming the phantoms are immersed in specific matching medium [84]. With this approach, retrieving simulation data for thousands of breast phantoms takes only one minute on a standard personal computer—compared to several days when running conventional simulations on high-performance GPU workstations. Beyond providing an ML-based simulation model, the authors also analyzed the relationship between phantom size and the minimum number of layers required in a CNN, offering physics-based insights into optimizing network architectures for this purpose.

## 7. Conclusions and Outlook

Microwave medical imaging, enhanced by ML, has shown great promise in advancing disease detection and diagnostic accuracy. DL models, particularly CNNs and U-Net architectures, have been widely explored for image reconstruction, artifact removal, and tissue characterization in applications such as breast cancer detection, brain imaging, and thermoacoustic imaging. While end-to-end learning systems have demonstrated effectiveness in mapping microwave scattering data to meaningful dielectric properties, challenges related to data scarcity and model generalization persist. Recent efforts have introduced generative models, such as GANs, to synthesize realistic training datasets, improving the robustness of ML-based reconstruction and classification. Additionally, hybrid imaging approaches, including the integration of microwave and ultrasound data, have been investigated to leverage complementary information for enhanced diagnostic performance. Despite these advancements, the practical implementation of ML-driven microwave imaging systems in clinical environments remains a challenge, requiring further validation, interpretability improvements, and computational efficiency enhancements.

Future research will focus on overcoming limitations such as training data availability and diversity, integrating physics information and conditions into ML models, and computational resources. As can be noticed, current ML models only adopt a few thousand pieces of simulation data or a few tens or hundreds of experimental data to conduct the training, leaving a considerable overfitting issue. Existing models may be improved by increasing data diversity through advanced generative models and domain adaptation techniques. Additionally, the integration of physics-informed NNs and explainable AI methods can enhance model interpretability and reliability, addressing concerns about black-box decision making in medical imaging. Introducing techniques such as SHapley Additive exPlanations (SHAP), the generalization of class activation mapping (Grad-CAM), and attention mechanisms can help enhance model transparency. Furthermore, hardware and computational costs are another challenge for developing enhanced ML-based microwave medical application models. Thus, optimizing computational efficiency through lightweight architectures and cloud-based processing solutions will be essential, especially for real-time clinical applications. Expanding clinical validation through large-scale patient studies and fostering interdisciplinary collaboration between engineers, medical professionals, and regulatory bodies will be critical for transitioning ML-based microwave imaging from research to clinical practice. The continued evolution of multimodal imaging approaches, combining microwave with established modalities such as MRI or CT, may further enhance diagnostic capabilities, paving the way for more accurate and accessible medical imaging solutions.

## Figures and Tables

**Table 1:** Comparison of ML application for classification.

Developers	Application	Algorithm	Accuracy	Training Data Resource
Hossain [24]	Brain	CNN	96.97%	Experiment
Gong [26]	Brain	DT	90%	Experiment
Singh [27]	Brain	Multiple	94%	Simulation
Ullah [28]	Brain	CNN	87%	Simulation
El-Shenawee [29]	Breast	NN	96.24%	Simulation
Franceschini [30]	Breast	CNN	96%	Simulation
Reimer [31]	Breast	CNN	73%	Experiment
Oliveira [33]	Breast	RF	unclear	Simulation
Mojabi [35]	Breast	CNN	Not apply	Simulation
Wang [36]	Breast	CNN	96.84%	Simulation
Rana [41]	Breast	SVM	91%	Simulation
Li [42]	Breast	CNN	93.2%	Experiment
Ozsobaci [43]	Breast	SVM	94.4%	Experiment
Geng [44]	Cold Pain	RF	93.75%	Experiment
Ruiz [45]	Neck	CNN	90%	Simulation
Cataldo [46]	Skin Cancer	CNN	unclear	Experiment

**Table 2:** Comparison of DL techniques for image reconstruction.

Developers	Application	Main NN Features	Training Data Resource
Cheng [55]	Brain	An NN for coarse reconstruction followed by a U-net for image quality improvement	Simulation
Abbosh [56]	Brain	A pretrained U-Net fine-tuned through transfer learning	Simulation
Kidera [63]	Breast	An encoding–decoding model with a two-step training procedure	Simulation
Baselice [66]	Breast	A fully connected NN trained by synthetic phantoms	Simulation
Bicer [70]	Breast	A fully connected NN followed by a U-net	Experimental scan
Dachena [71]	Neck	Fully connected NN	Simulation

## Data Availability

No new data were created or analyzed in this study.
